# Impacts of a prolonged marine heatwave and chronic local human disturbance on juvenile coral assemblages

**DOI:** 10.1371/journal.pone.0300084

**Published:** 2025-02-25

**Authors:** Kristina L. Tietjen, Nelson F. Perks, Niallan C. O’Brien, Julia K. Baum

**Affiliations:** 1 Department of Biology, University of Victoria, Victoria, British Columbia, Canada; 2 Hawaii Institute of Marine Biology, Kaneohe, Hawaii, United States of America; The University of Auckland - City Campus: University of Auckland, NEW ZEALAND

## Abstract

Coral reefs are threatened by climate change and chronic local human disturbances. Although some laboratory studies have investigated the effects of combined stressors, such as nutrient enrichment and heat stress, on growth and survival of early life stage corals, *in situ* studies remain limited. To assess the influence of multiple stressors on juvenile corals, we quantified densities of corals ≤ 5 cm at 18 forereef sites with different exposure levels to underlying chronic local human disturbance before, during, and after the 2015-2016 El Niño. This marine heatwave caused prolonged heat stress and devastating losses of coral cover on the shallow forereef’s of Kiritimati, in the central equatorial Pacific Ocean. Here, we enumerated a total of 7732 juvenile corals from 13 different families. Over 80% of corals were from four families: 70% from Agariciidae, Merulinidae, or Poritidae, which all have stress-tolerant life history strategies, and 11% from Acroporidae which has a competitive life-history strategy. Both local disturbance and heat stress were significantly negatively related to juvenile coral densities. Prior to the heatwave, juvenile densities were on average 72% lower at the most disturbed sites (7.2 ±  1.9 m^-2^) compared to the least disturbed ones (15.3 ±  3.8 m^-2^). Overall, juvenile corals had a lower bleaching prevalence and lower mortality during the heatwave when compared to their adult counterparts. Still, the heatwave resulted in the loss of half (49%) of all juvenile corals, with those corals with competitive or weedy life history strategies undergoing greater declines than stress-tolerant ones. Although juvenile coral densities increased slightly in the year following the heatwave, the effect was statistically non-significant. Our results highlight the influence of chronic local anthropogenic and marine heatwaves on juvenile coral densities.

## Introduction

Coral reefs are increasingly impacted by the effects of climate change, which are overlaid on chronic regional and local scale anthropogenic pressures [1,2]. Recent marine heatwaves, notably the 2015-2016 El Niño, have caused mass coral bleaching and extensive mortality [3–5]. The persistence of coral reefs is dependent on the recovery of corals, which can be driven by coral recruitment and the population dynamics of juvenile corals [6–8]. But while heatwave impacts have been documented extensively for adult corals, there has been comparatively less research on the effects of heat stress on juvenile corals. Previous studies have demonstrated that the demographics of juvenile corals can strongly influence recovery trajectories [6,7,9], with surviving juvenile corals rising through size classes, with corresponding increases in brood stock and reproductive output [10], to repair stock-recruitment relationships disrupted by the loss of adult colonies [10,11]. Studies of long-term reef recovery following the 1998 El Niño in the Indian Ocean (Scott Reef, Western Australia and the Maldives) found that juvenile corals stimulated reef recovery within 10-12 years of the event [10,12,13], and were likely especially critical on these isolated reefs that are reliant on self-seeding [10,14]. Overall, however, given their importance for recovery dynamics, and the lack of studies, there is a need to better understand the effects of climate change-amplified heat stress, and its interplay with local anthropogenic disturbance, on juvenile corals.

Although studies from several regions, including the Caribbean, Mediterranean, Japan, Thailand, Indonesia, and Australia [15–19], have shown that juvenile corals can have greater heat stress tolerance than their conspecific adults, the reasons underlying this difference remain unclear [15–17]. Mumby [15] hypothesized that this enhanced survival could be due to reduced irradiance levels due to their cryptic microhabitats or capacity for heterotrophic feeding to replace lost autotrophic nutrition during bleaching. Further research has suggested additional mechanisms based on properties such as being non-reproductive, which may allow for more energy invested into maintenance [18], and the relatively flat [20] and small colonies of juveniles, which may allow for faster elimination of toxic by-products by mass transfer [21]. In addition to exploring mechanisms for survival, a few studies have investigated the effects of heat stress on juvenile corals. Two experimental laboratory studies investigated the effects of short term heat stress and found that it resulted in sub-lethal stress and negative allometric growth scaling in *Porites* [22,23]. A third experiment, which examined heat stress as well as the influence of a simulated river plume and terrestrial runoff nutrient enrichment event on 4-month-old *Acropora* corals, found that while heat stress alone led to increases in growth and mortality, when both stressors were present juvenile mortality was reduced, suggesting that nutrient enrichment can lessen the effects of thermal stress [24].

Juvenile survival through heat stress [7,18], and contributions to reef recovery through increases in coral cover [7], vary substantially amongst coral species and life history types. For example, a study by Doropoulos and colleagues [7] on the Great Barrier Reef, Australia found that when reef recovery is characterized by increases in coral cover, brooders may not contribute substantially to reef recovery due to their small colony sizes [7,25] despite their other ‘weedy’ life history characteristics (e.g., rapid generation times, opportunistically colonizing recently disturbed habitats) [25,26]. Rather, corals such as *Acropora*, that exhibit a ‘competitive’ life history strategy, grow large colonies, and excel at colonizing [25] can contribute considerably to coral cover [7], and thus play a major role in reef recovery. In comparison, massive corals that have a ‘stress-tolerant’ life history strategy often survive disturbance events [25], but do not contribute appreciably to increases in coral cover because of their slow growth rates [7].

Here we aimed to advance understanding of how heat stress, when combined with local anthropogenic stressors, affects the densities of juvenile corals and how these impacts vary across corals with different life history strategies. To do so, we capitalized on a prolonged marine heatwave that occurred on Kiritimati (Christmas Island) during the 2015-2016 El Niño, which was overlaid on the atoll’s gradient of chronic local anthropogenic disturbance [27,28]. Kiritimati is geographically isolated, and thus is largely reliant on self-seeding for coral recruits [29,30]. We censused juvenile corals via video assays at 18 sites along the disturbance gradient before, during, and one year after the El Niño. This ecosystem-scale natural experiment allowed us to examine the impacts of prolonged heat stress on juvenile corals at sites exposed to different intensities of local human disturbance, and its effect on an isolated island’s initial reef recovery. With respect to juvenile coral densities around the atoll prior to the heatwave, we hypothesized that: (*i*) due to the lower overall coral cover and reef structural complexity [31] at sites with high disturbance these sites would also have reduced overall juvenile coral densities, and (*ii*) that this would include reduced densities of competitive and stress-tolerant juvenile corals, but that due to weedy coral’s propensity to colonize disturbed areas, juvenile corals with this life history strategy would have greater densities at more highly disturbed sites. We also hypothesized that the heatwave would result: (*iii*) in increased juvenile coral bleaching around the island, and that corals at sites with the highest local human disturbance would have the greatest prevalence of bleaching due to pre-existing stressors; (*iv*) in a significant decline in juvenile coral densities around the atoll due to the prolonged heat stress; and (*v*) that sites with the lowest local human disturbance would suffer the greatest juvenile coral losses, given that corals at high disturbance sites would have likely already declined substantially; and (*vi*) that mortality would vary amongst coral life history strategies, with the highest survival in stress-tolerant juvenile corals, the lowest survival in competitive corals because of their sensitivity to thermal stress, and the greatest density increases in the year following the heatwave in weedy corals because of their fast growth rates.

## Materials and methods

### Study area and design

**Fig 1:**
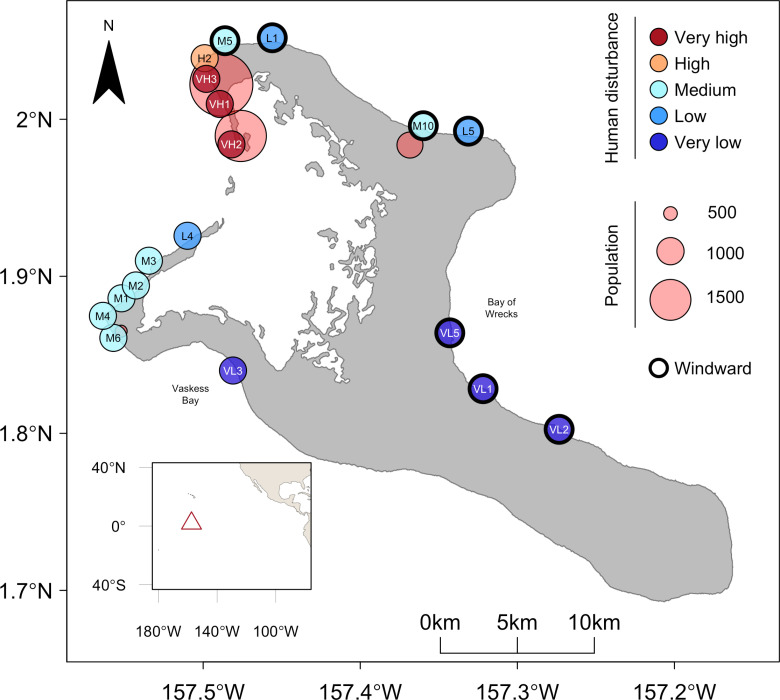
Map of shallow forereef study sites on Kiritimati (Christmas Island), categorized by level of chronic local human disturbance. Village populations (red circles) are represented by bubble size and windward sites are denoted by a thick black border around site. Inset shows Kiritimati’s location in the equatorial central Pacific Ocean (open triangle).

We quantified juvenile coral density by surveying their densities before, during, and after the 2015-2016 El Niño-induced heatwave, at eighteen shallow forereef (10-12 m isobath) sites spanning a gradient of local human disturbance on Kiritimati (Christmas Island, Republic of Kiribati). Kiritimati, a remote coral atoll in the central equatorial Pacific Ocean (01°52’N, 157°24’W), is the world’s largest atoll by land mass (388 km^2^; 150 km in perimeter; Fig 1). Sites were surveyed at three time points prior to impacts of the El Niño (two years before (July 2013; 16 sites), 1 year before (August 2014; 9 sites), and at the beginning (May 2015; 8 sites)), then resurveyed two months (early, July 2015; 13 sites) and 10 months (late, March 2016; 11 sites) into the heatwave, and again approximately 14 months after the end of the heatwave (1 year after; July 2017; 18 sites) (S1 Fig and S1 Table). Although not all sites were surveyed at each time point due to inclement weather conditions that prevented safe boat and/or diving access, the full set of eighteen sites were surveyed at least once before and once after the heatwave for direct comparability. The field research was conducted with permission from the Government of the Republic of Kiribati through permit numbers 008/13, 007/14, 001/16, 003/17. Information regarding the ethical, cultural, and scientific considerations specific to inclusivity in global research is included in the Supporting Information (S1 Checklist).

Chronic local human disturbance around the island was previously quantified [27,32] using a combined quantitative metric that incorporated both the human population within 2 km radius [33] of each site (as a proxy for localized impacts, e.g., sewage inflow) [27,32] and fishing intensity data [28]. We modelled local human disturbance using this continuous metric, and for visualization used five local human disturbance categories (assigned previously from the continuous metric), which should be regarded as being relative to other sites rather than absolute levels of human disturbance [27]. Thermal stress, in degree heating weeks, was quantified from *in-situ* temperature data, collected from temperature loggers (SBE 56, Sea-Bird Scientific ±  0.001°C precision) deployed at monitored sites around the island, with at least one logger in each of the disturbance levels [further details in 37]. The heat stress from the 2015-2016 El Niño peaked at 27 Degree Heating Weeks (DHW) on Kiritimati, and temperatures were continuously elevated between June 2015 and April 2016 [34] with minimal differences in heat stress across sites ( < 1.1 DHW) [27,34]. This prolonged heat stress resulted in an 89% loss of coral cover across the atoll [27].

In addition to local disturbance, oceanographic factors also vary around the atoll. We used site level net primary productivity (NPP; mg C m^-2^ day^-1^) data extracted from the Marine Socio-Environmental Covariates (MSEC) open source data product, derived from NOAA CoastWatch and calculated over a 2.5 acrmin grid (https://shiny.sesync.org/apps/msec/ [35]). In lieu of comprehensive site-level wave exposure data, we defined site-level exposures based on the dominate wind direction (southeasterly [36]), with sheltered sites on the west side of the island grouped as leeward, and exposed sites on the north and east coasts as windward, following [27,32,37] (Fig 1).

### Juvenile coral survey

**Fig 2:**
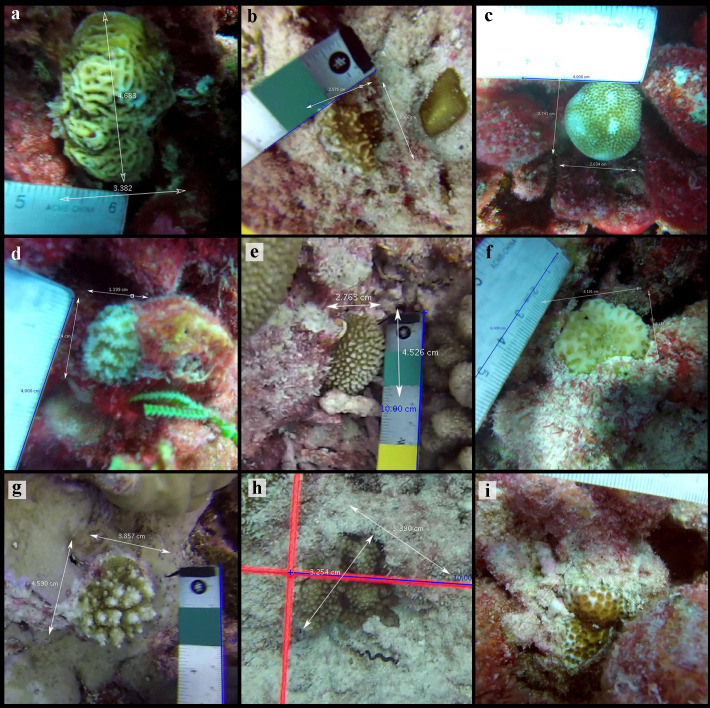
Representative photos of surveyed juvenile corals on Kiritimati throughout the study. The top three most common corals were **(a)** *Leptoseris mycetoseroides*, **(b)** *Pavona varians*, and **(c)** *Porites lobata*. Panels d-f (d =  *Platygyra* spp., **e** =  *Hydnophora microconos*, **f** =  *Goniastrea stelligera*) are examples of other stress-tolerant corals. Panels **g** (*Acropora* spp.) and **h** (*Pocillopora* spp.) are examples of corals with competitive life histories, and panel i (*Leptastrea purpurea*) has a weedy life history strategy. Photos: Baum Lab, University of Victoria.

At each site, we surveyed juvenile corals along two 25 m transects. Up to ten 1 m^2^ gridded quadrats were set at predetermined random points along the transects and filmed following the protocol in Mumby and colleagues [38], with the modification of 10 cm swaths instead of 20 cm (n =  732 videos total, S1 Table and S2 Fig); more than ten quadrats were surveyed at site VH1 to account for the high prevalence of sand at that site. Videos were randomly analyzed by one of three trained individuals (KLT, NFP, NCOB), who identified each coral to the lowest possible taxonomic unit and measured its 2D horizontal size (i.e., widest width) using the software Tracker (physlets.org/tracker/ [39]; Fig 2); KLT also checked identifications to ensure consistency amongst observers. A juvenile coral was distinguished as being a new colony—rather than a fragment of a colony that experienced partial mortality—by inconsistencies with the surrounding area (i.e., dead skeleton). We defined juvenile corals as those with ≤ 5 cm maximum width. While this size classification is somewhat arbitrary in regards to maturity of corals, it is a commonly used size classification in juvenile and young coral studies [7,18,19,40,41]. Corals that were not fully inside a quadrat were removed from the dataset.

We categorized each identified juvenile hard coral as stress-tolerant, competitive, weedy, or generalist based upon their life history traits using the Coral Trait Database (https://coraltraits.org [25]), and consistently with Baum and colleagues [27] (S2 Table). Three coral taxa (n =  42 individuals total) that could not be assigned to a life history strategy were removed from statistical analysis. In addition, corals with unresolved identifications or life history assignments (e.g., when coral could only be identified to family and a life history could not be assigned to the family due to multiple strategies within the family) were removed from statistical analysis (n =  317; 4.1% of the data). Generalists (n =  5) and soft coral (n =  38) were excluded from our statistical analysis (apart from species specific Wilcoxon tests) due to low densities.

### Statistical analyses

All statistical analyses were conducted in R v.4.4.1 [42]. GLMMs were run using the package *glmmTMB* [43]. Wilcoxon tests were run using R’s base *stats* package [42]. Prior to analysis, all continuous input variables were standardized to a mean of zero and a standard deviation of 0.5 using the ‘rescale’ function in the *arm* package [44]. AIC was used in model selection and diagnostic graphs plotting residuals using the *DHARMa* package [45] were analyzed for each model presented. For each model type, we present the top model according to that with the lowest AIC value. Figures are presented using the categorical variable for local human disturbance as in [27]. All data and code that support the results of this study are available on GitHub (https://github.com/baumlab/Tietjen_etal_2025_PlosOne) with the following identifier: [https://doi.org/10.5281/zenodo.14585412].

#### Pre-heatwave analyses (hypotheses *i* and *ii*).

We first examined how juvenile coral densities varied around the atoll prior to the heatwave, by fitting generalized linear mixed-effects models (GLMMs) to the data for the three pre-heatwave timepoints, using a negative binomial error structure to accommodate overdispersion in the data. We included an interaction between local human disturbance (continuous) and life history strategy (categorical), along with reef exposure (leeward, windward) and NPP, as fixed effects, while site was modelled as a random effect. Local human disturbance was modelled using a quadratic relationship to allow for the possibility of a non-linear relationship. We also included an offset to account for differences in the total number of quadrats sampled at each site. Originally, we included year as a factor in the model, but since it did not influence overall results or improve the AIC it was dropped.

#### Heatwave effects: juvenile coral bleaching analyses (hypothesis *iii*).

To test if the proportion of bleached juvenile corals changed across the local human disturbance gradient and throughout the heatwave, we fit a GLMM to the data using a weighted binomial structure. We included an interaction between local human disturbance (continuous) and heat stress (before, early, late time periods), along with reef exposure (leeward, windward) and NPP, as fixed effects, while site was modelled as a random effect. Local human disturbance was modelled using a quadratic relationship to allow for the possibility of a non-linear relationship. We also included an offset to account for differences in the total number of quadrats sampled at each site.

#### Heatwave effects: juvenile coral density analyses (hypotheses *iv* – *vi*).

We then tested for the influence of the heatwave on juvenile coral density by adding to the pre-heatwave models ‘heat stress’ as an additional fixed effect (four levels: before, early, late, and after the heatwave) and a two-way interaction between ‘heat stress’ and local disturbance (modelled as a quadratic) to examine if the heatwave’s impact was modulated by local disturbance. We conducted two sensitivity analyses to test if the ‘heat stress’ effect was dependent on the exact sites sampled in the various expeditions. The first sensitivity analysis included only the 9 sites that were sampled in all four periods and the second included the 10 sites sampled in both the before and in the late heat stress time periods (S3 Table and S1 Fig).

We also investigated if the impacts of the heatwave varied with coral life history strategy, by fitting separate ‘heat stress’ models, as above, for each of the three main life history types. Finally, to test for significant differences in juvenile coral density before and after the heatwave for each species, we used Wilcoxon matched-pairs signed-rank tests as the data was not normally distributed.

## Results

**Fig 3:**
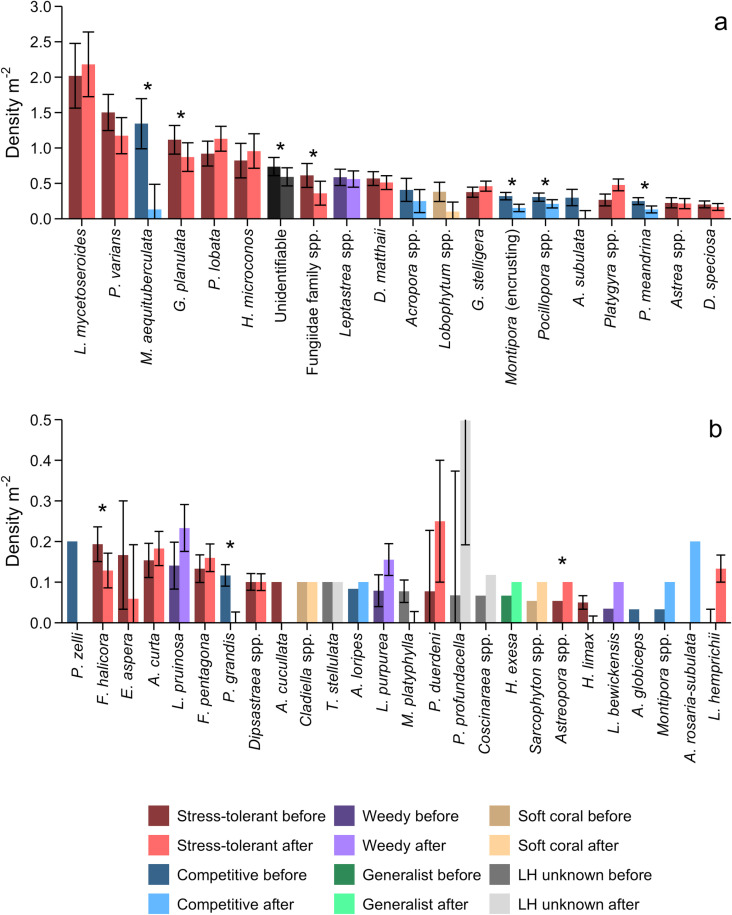
Density ( ± SE) of 45 juvenile (≤5 cm) coral species (or genus) before (2013, 2014, and 2015b) and after (2017) the marine heatwave. Plots **(a)** 20 most common and (b) rarer corals are colored by life history and heatwave period ( * =  significantly different at α =  0.05, S5 Table). Error bar for *P. profundacella* (in panel b) after the marine heatwave extends to 0.8042 m^-2^. Y-axis scales vary between plots.

**Fig 4:**
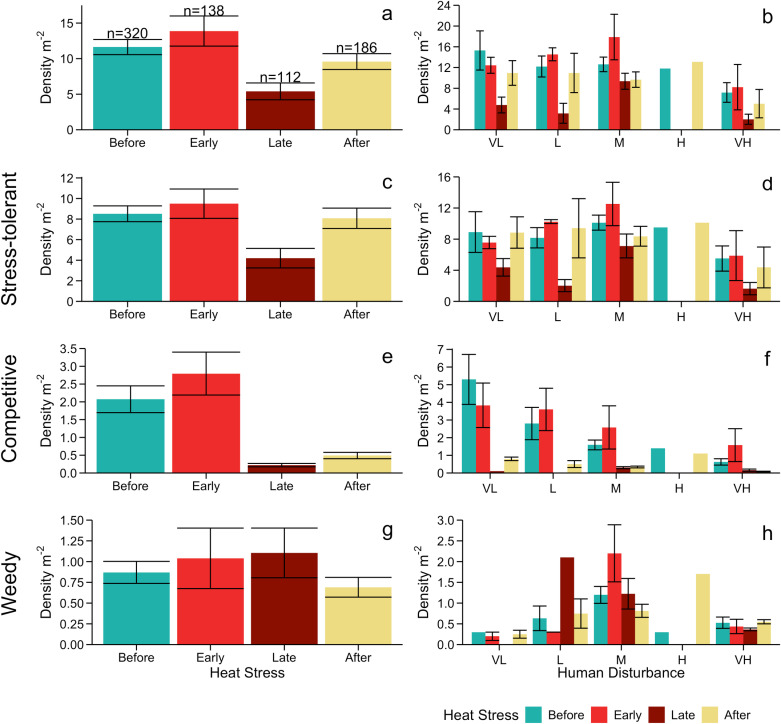
Density (± SE) of juvenile (≤5 cm) corals at forereef sites on Kiritimati (Christmas Island) across the heatwave. (Before =  pooled July 2013, August 2014, May 2013, Early =  2 months (July 2015), Late =  10 months (March 2016), and After =  ~ 1 year after (summer 2017)) for **(a, b)** all corals, **(c, d)** stress-tolerant corals, **(e, f)** competitive corals, and **(g, h)** weedy corals across the **(a, c, e, g)** entire island (18 sites; n =  the number of quadrats sampled per time point), and the **(b, d, f, h)** local human disturbance gradient (VL =  very low, L =  low, M =  medium, H =  high, and VH =  very high). Note: Not all sites could be sampled in each time point. Y-axis scale varies among panels.

In total, we enumerated 7732 juvenile corals (n =  7320 hard; n =  38 soft; n =  374 unidentifiable) from 732 census quadrat videos. Juvenile corals had an overall mean density of 10.7 m^-2^ ( ± 0.74 SE) and a mean width of 2.28 cm ( ± 0.01 SE; S3 Fig). We identified 45 juvenile coral species (or genera when species was not possible) from 13 different families, the most common of which were *Leptoseris mycetoseroides* (2.1 ±  0.08 m^-2^), *Pavona varians* (1.3 ±  0.16 m^-2^), and *Porites lobata* (1.0 ±  0.10 m^-2^) (Figs 2 and 3). Over 80% of enumerated corals belonged to four families: Agariciidae (n =  2732), Merulinidae (n =  1874), Acroporidae (n =  873), and Poritidae (n =  774). Stress-tolerant corals were the most common at every time point and accounted for almost three-quarters (73%) of the corals overall (n =  5661) (Figs 3 and 4). Competitive corals were the next most common (n =  1065 corals; 13.8%), followed by weedy (n =  550, 7.1%), and generalists (n =  5) (Fig 4).

### Pre-heatwave juvenile corals (hypotheses *i* and *ii*)

Prior to the heatwave, overall mean juvenile coral density ranged from 7.2 m^-2^ ( ± 1.9 SE) at the most disturbed sites up to 15.3 m^-2^ ( ± 3.8 SE) at the least disturbed ones (Fig 4). Accordingly, and in agreement with hypothesis *i*, pre-heatwave juvenile coral density was significantly negatively influenced by local human disturbance (Table 1). Juvenile coral densities also varied significantly with life history strategy and there was a significant interaction between local human disturbance and life history strategy (Table 1). Densities of both competitive and weedy juvenile corals were significantly less common than stress-tolerant corals (Table 1). As hypothesized (*ii*), densities of both stress-tolerant and competitive corals declined significantly as local human disturbance increased, however the highest density of weedy corals was at sites with moderate levels of local human disturbance, although this trend was not significant (S4 Fig.).

**Table 1:** Results for final pre-heatwave models. Bolded values are significantly different from baseline levels (i.e., stress-tolerant, leeward) at α =  0.05. Red shaded boxes correspond to variables with a negative parameter estimate.

		Parameter estimate	Std. error	Z value	P
Human disturbance	Linear	**-2.936**	**1.227**	**-2.393**	**0.017**
	Quad.	-1.194	0.940	-1.271	0.204
HD * LH (Competitive)	Linear	**-3.163**	**1.397**	**-2.264**	**0.024**
	Quad.	**4.412**	**1.207**	**3.655**	**<0.001**
HD * LH (Weedy)	Linear	3.408	1.741	1.957	0.050
	Quad.	-2.343	1.702	-1.377	0.169
Life history	Competitive	**-1.545**	**0.131**	**-11.838**	**<0.001**
	Weedy	**-2.493**	**0.182**	**-13.735**	**<0.001**
Exposure	Windward	0.143	0.227	0.629	0.529
Net Primary Productivity		0.124	0.2607	0.477	0.633

Quad. = Quadratic; HD = Human disturbance; LH = Life history

### Heatwave effects: juvenile coral bleaching (hypothesis *iii*)

**Fig 5:**
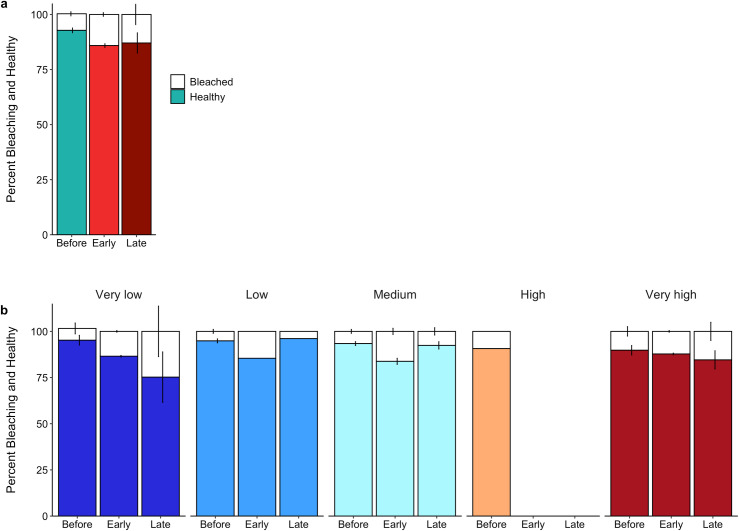
Percent of juvenile corals visually bleaching or healthy across the (a) marine heatwave and (b) the human disturbance gradient. Heat stress colors correspond with figure 4 and human disturbance colors correspond with figure 1.

The heatwave caused significant increases in the percentage of juvenile coral colonies that were bleached above background levels (Fig 5a and S5 Fig), with the largest effect occurring early in the heatwave (Table 2). Indeed, many coral species showed an increase from low background bleaching levels early in the heatwave to decreases late in the event (S4 Table). For example, *L. mycetoseroides*, the most common juvenile coral, had 3.4% bleaching before the heatwave, which increased to 25.3% early on and then decreased to 9.8% by the end of the heatwave (S4 Table). And while prior to the heatwave there was a non-significant positive relationship between the prevalence of bleaching in juvenile corals and local human disturbance, by two months into the heatwave (early time point) it was significantly higher in colonies located at lower local disturbance sites, and by late in the event, those in the very lowest and the very highest disturbed areas had the highest prevalence of bleaching, albeit not significantly (Table 2 and Fig 5b and S5 Fig).

**Table 2:** Results for overall bleaching model. Bolded values are significantly different from baseline levels (i.e., before, leeward) at α =  0.05. Red shaded boxes correspond to variables with a negative parameter estimate.

		Parameter estimate	Std. error	Z value	P
Human disturbance	Linear	1.083	0.680	1.59	0.111
	Quad.	-0.320	0.604	-0.53	0.596
Heat stress	Early	**1.691**	**0.112**	**15.05**	**<0.0001**
	Late	**1.151**	**0.171**	**6.74**	**<0.0001**
HD * HS (Early)	Linear	**-2.276**	**0.807**	**-2.82**	**0.0048**
	Quad.	-1.174	0.851	-1.38	0.168
HD * HS (Late)	Linear	-1.591	1.200	-1.35	0.177
	Quad.	2.116	1.226	1.73	0.084
Exposure	Windward	0.202	0.185	1.09	0.274
Net Primary Productivity		-0.004	0.256	-0.02	0.986

HD = Human disturbance; Quad. = Quadratic relationship; HS = Heat stress

### Heatwave effects: juvenile coral densities (hypotheses *iv*-*vi*)

**Fig 6:**
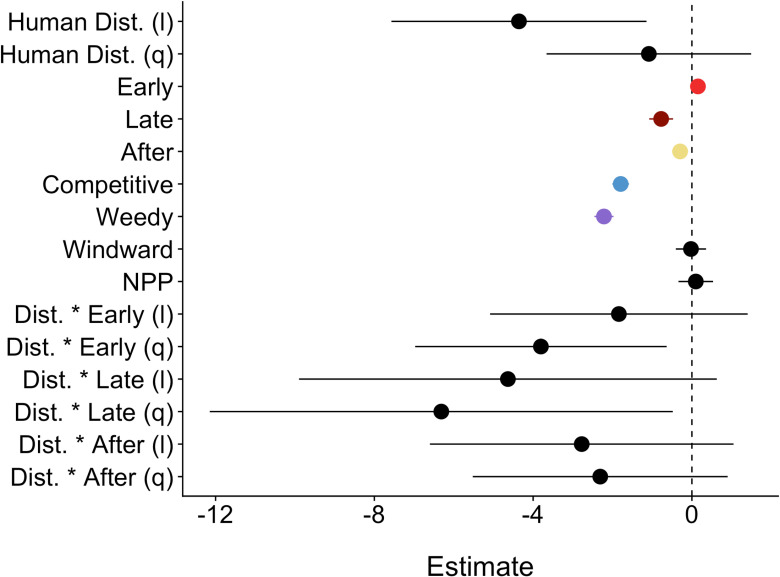
Generalized linear mixed model predictor coefficient effect size estimates and 95% confidence intervals for the overall dataset throughout the heatwave. Human disturbance was modelled as a quadratic (l = linear, q =  quadratic). Heat stress colors correspond with figure 4 and life history with figure 3.

Overall, as hypothesized (*iv*), the heatwave significantly reduced juvenile coral densities (Table 3). Although no losses were detected two months into the heatwave, by late in the event half (49%) of all juvenile corals had been lost (Figs 4a and 6). While absolute losses of juvenile coral densities were greater at very low disturbance sites, relative losses were comparable between sites exposed to either very low (68.9%) or very high (66.5%) disturbance; relative losses were much less at sites with medium disturbance (20.6%) (Fig 4b). We had hypothesized (*v*) that declines would be greatest at very low disturbance sites, but did not anticipate that instead of tailing off, declines would increase again at very high disturbance sites (S6 Fig). This altered relationship between juvenile coral density and disturbance meant that the interaction between local disturbance (modelled as a quadratic) and heat stress period was significant (Table 3 and S6 Fig). A year after the heatwave, juvenile corals densities had increased overall, but still remained significantly lower than they had been prior to the event (Figs 4a and 6 and Table 3). In our sensitivity analyses that controlled for sites sampled at each time point, all of these patterns held (S3 Table).

**Table 3:** Results for final heatwave juvenile coral density model. Bolded values are significantly different from baseline levels (i.e., before, stress-tolerant, leeward) at α =  0.05. Red shaded boxes correspond to variables with a negative parameter estimate.

		Parameter estimate	Std. error	Z value	P
Human disturbance	Linear	**-4.357**	**1.640**	**-2.657**	**0.008**
	Quad.	-1.085	1.315	-0.825	0.409
Heat stress	Early	0.152	0.098	1.555	0.120
	Late	**-0.774**	**0.154**	**-5.042**	**<0.0001**
	After	**-0.298**	**0.103**	**-2.892**	**0.004**
HD * HS (Early)	Linear	-1.840	1.656	-1.111	0.267
	Quad.	**-3.804**	**1.617**	**-2.353**	**0.019**
HD * HS (Late)	Linear	-4.635	2.686	-1.726	0.084
	Quad.	**-6.314**	**2.976**	**-2.121**	**0.034**
HD * HS (After)	Linear	-2.776	1.951	-1.422	0.155
	Quad.	-2.310	1.639	-1.409	0.159
Life history	Competitive	**-1.794**	**0.107**	**-16.811**	**<0.001**
	Weedy	**-2.216**	**0.123**	**-18.076**	**<0.001**
Exposure	Windward	-0.0237	0.195	-0.122	0.903
Net Primary Productivity		0.098	0.222	0.440	0.660

HD =  Human disturbance; LH =  Life History; Quad. =  Quadratic relationship; HS =  Heat stress

**Fig 7:**
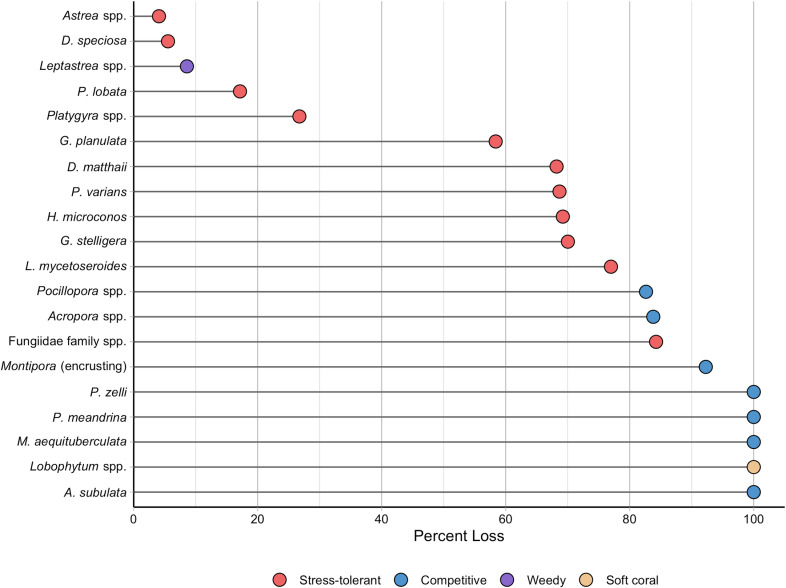
Overall change in density by the late heat stress time period of individual juvenile (≤ 5 cm) coral taxa at forereef sites on Kiritimati (Christmas Island) for the 20 most common taxa before the heatwave, ordered from least to greatest change in density. Life history colors correspond with Fig 3.

The heatwave’s effects varied depending on juvenile coral life history strategy. Both competitive and stress-tolerant corals declined significantly (Table 4 and S7 Fig), but losses were much greater for competitive corals (96.8%) than stress-tolerant ones (69.3%), as hypothesized (*vi*); changes in weedy coral densities were not statistically significant (Table 4 and S7 Fig). Indeed, of the 20 coral taxa with the highest pre-heatwave densities, all seven with competitive life histories declined by over 80% (four by 100%), while there was a greater range of losses among the stress-tolerant taxa: only species from the Fungiidae family had losses exceeding 80% while four other taxa had declined by less than 50% (Fig 7). How human disturbance modulated the impact of the heat stress differed amongst life history strategies: stress-tolerant corals had a significant quadratic relationship with human disturbance early in the heatwave (mirroring the pattern described for juvenile corals above) that shifted to a linear relationship by the late time period (Table 4 and S7 and S8 Fig). Competitive coral densities also had a significant quadratic relationship with human disturbance early in the heatwave but none of the following time periods had this significant relationship, likely due to the huge overall decline in density (Table 4 and S7 and S8 Fig). In contrast, weedy corals had a significant linear relationship late in the heatwave (S7 and S8 Fig). A year after the heatwave, densities of stress-tolerant juvenile corals had increased to the extent that they were not statistically different from their densities before the event (Table 4 and Fig 4 and S7 Fig); one stress-tolerant taxa even exhibited a small but statistically significant increase (Fig 3 and Table S5). In contrast, while competitive coral densities had increased slightly, they remained significantly lower than before the heatwave (Table 4 and Fig 4 and S7 Fig). Moreover, our comparison of the densities of individual taxa from before to one year after the heatwave showed that most of those still exhibiting significant decreases had competitive life histories (Fig 3 and S5 Table).

**Table 4:** Results for (a) stress-tolerant, (b) competitive, and (c) weedy life history strategy heatwave models. Bolded values are significantly different from baseline levels (i.e., before, leeward) at α =  0.05. Red shaded boxes correspond to variables with a negative parameter estimate.

(a) Stress-tolerant					
	Parameter estimate		Std. error	Z value	P
Human disturbance	Linear	**-2.487**	**1.077**	**-2.309**	**0.021**
	Quad.	-0.888	0.852	-1.042	0.297
Heat stress	Early	0.0878	0.106	0.832	0.405
	Late	**-0.594**	**0.149**	**-3.990**	**<0.0001**
	After	-0.124	0.102	-1.219	0.223
HD * HS (Early)	Linear	-1.233	1.092	-1.129	0.259
	Quad.	**-2.301**	**1.044**	**-2.204**	**0.028**
HD * HS (Late)	Linear	**-3.088**	**1.565**	**-1.973**	**0.049**
	Quad.	-1.965	1.561	-1.259	0.208
HD * HS (After)	Linear	-1.350	1.134	-1.190	0.234
	Quad.	-1.103	0.943	-1.169	0.242
Exposure	Windward	0.021	0.218	0.097	0.923
Net Primary Productivity		0.040	0.250	0.161	0.872
**(b) Competitive**					
Human disturbance	Linear	**-5.095**	**1.524**	**-3.344**	**<0.001**
	Quad.	1.993	1.148	1.736	0.083
Heat stress	Early	**0.345**	**0.162**	**2.125**	**0.034**
	Late	**-2.195**	**0.471**	**-4.660**	**<0.0001**
	After	**-1.606**	**0.260**	**-6.167**	**<0.0001**
HD * HS (Early)	Linear	1.501	1.546	0.971	0.331
	Quad.	**-2.917**	**1.380**	**-2.114**	**0.035**
HD * HS (Late)	Linear	0.705	4.672	0.151	0.880
	Quad.	-9.117	5.730	1.591	0.112
HD * HS (After)	Linear	1.216	2.705	0.450	0.653
	Quad.	-2.054	2.236	-0.918	0.358
Exposure	Windward	0.664	0.299	2.224	0.262
Net Primary Productivity		0.319	0.372	0.857	0.391
**(c) Weedy**					
Human disturbance	Linear	-2.615	1.395	-1.874	0.061
	Quad.	-1.892	1.321	-1.433	0.152
Heat stress	Early	0.340	0.236	1.440	0.150
	Late	-0.720	0.457	-1.575	0.115
	After	-0.157	0.240	-0.655	0.512
HD * HS (Early)	Linear	-4.826	2.576	-1.874	0.061
	Quad.	-4.285	2.806	-1.527	0.127
HD * HS (Late)	Linear	**-8.700**	**4.413**	**-1.971**	**0.0487**
	Quad.	-11.556	6.342	-1.822	0.068
HD * HS (After)	Linear	-4.094	2.690	-1.522	0.128
	Quad	-3.104	2.502	-1.241	0.215
Exposure	Windward	**-1.158**	**0.309**	**-3.750**	**0.0002**
Net Primary Productivity		0.469	0.282	1.663	0.096

HD = Human disturbance; Quad. = Quadratic relationship; HS = Heat stress

## Discussion

Our study provides evidence that both chronic local anthropogenic disturbance and prolonged heat stress significantly reduce the density of juvenile corals on shallow forereefs. Before the heatwave, there was an inverse relationship between juvenile coral densities and the local disturbance gradient overall, but varying impacts of disturbance on different coral life histories. The heatwave significantly reduced juvenile coral densities, and for each life history strategy shifted the relationship between their density and local human disturbance. Juvenile coral densities appeared to be increasing (by > 50%) just one year after the heatwave, but the differences were not statistically significant.

The significant negative effect of local human disturbance on juvenile coral densities we detected is in accordance with previous studies that have documented the negative effects single [24,46,47] and multiple [48] chronic anthropogenic stressors have on juvenile corals. On Kiritimati, this effect is believed to be the result of poor water quality, due to sewage and other pollution outflow onto the reef [27,28,49], and direct damage from dredging at some sites [27], which had substantially decreased overall coral cover and habitat complexity at the most disturbed sites prior to the heatwave [27]. We note that prior to the heatwave, turf algae and sediment cover were highest at the most disturbed sites, all of which can negatively impact corals of all life stages [22,50,51]. Comparatively, however, examining the pre-heatwave coral communities, the influence of human disturbance was greater on adults [27] than it was on juveniles (this study), although the mechanism for this difference remains unclear.

The 2015-2016 El Niño caused significant mortality of juvenile corals on Kiritimati, but this effect was only evident at our end of heatwave expedition (i.e., after 10 months of heat stress). Juvenile corals have been known to survive a few months of heat stress [15–17,52] which is hypothesized to be due to their small and flat structure increasing mass transfer of toxic by-products [20,21] and being non-reproductive [18]. Despite the apparent resilience of juvenile corals to short term heat stress, our study and others demonstrate juvenile coral’s susceptibility to long term elevated temperature conditions, similar to adult colonies [4,19,27,53]. Studies in the Seychelles documented 48% or greater loss of juvenile corals following the 2015-2106 El Niño [48,54] and a study in the Maldives recorded similarly low densities as our study (2.7 ±  4.6 to 5.8 ±  12.3 individuals m^-2^ [13];), and although they did not have pre-heatwave data, as mentioned by Perry and Morgan [13], in the context of densities reported after the 1998 El Niño [55,56] these are very low. In West Sumatra Province, Indonesia, the decline in juvenile corals was only 26% from before the El Niño to two years after (2018) [19]. On Kiritimati, recovery of juvenile corals may have occurred, as evidenced by the ability of reefs on Moorea to recover despite multiple acute disturbances resulting in low juvenile coral densities [57], however subsequent monitoring has been limited due to COVID-19 related international travel bans from 2020 to 2023.

A comparison between the heatwave impacts we documented here to those documented for adult corals by Baum et al. [27] on Kiritimati, reveals that the heatwave had a greater impact on adult corals, with a higher occurrence of bleaching and 1.8 fold greater mortality in adult colonies [27]. Before the 2015-2016 El Niño, Kiritimati juvenile corals as a whole were more frequently bleached than the adults [27], which could indicate that they were competing with post-settlement stressors as the escapement size, irrespective of life-history and habitat, is 5 cm [7]. However, during the heatwave, the bleaching frequency in adults surpassed that of the juveniles (S4 Table) [27], possibly due to juvenile corals’ hypothesized better resistance to bleaching [15,17,18,20]. In an Australian study, Álvarez-Noriega et al. [18] also found an overall difference in bleaching induced mortality between adults and juvenile corals, but that it was taxon dependent. This seems to be location dependent however, as we only found *Goniastrea* spp. to have the same trend on Kiritimati where the adults were more affected. The biggest difference was in the Merulinidae family, where in Australia the juveniles did worse than their adult counterparts, but on Kiritimati, the majority of the Merulinidae juveniles increased in density while the adults declined [27]. Small colonies of *Oculina patagonica* also had higher survivorship than their adult counterparts during a bleaching event in the Mediterranean [52], whereas in the inner Seychelles, Dajka and colleagues [48] documented 70% mortality of juveniles due to heat stress which was similar to the adult community loss on those reefs.

It was expected that corals at sites exposed to very high local disturbance before the heatwave would have been the most stressed and therefore have the highest prevalence of bleaching, however, before the heatwave bleaching prevalence was statistically similar across the island. This may suggest that the local human disturbance does not greatly exceed or add to that of post-settlement stressors. Interestingly, while there was a large decline in juvenile corals by the late time period across the atoll, the sites that had some of the highest bleaching percentages (medium disturbed sites) at the early time point had the smallest decline (~21%). Additionally, although bleaching prevalence 10 months into the heatwave (late time period) was highest at the very high and low disturbed sites, these sites saw larger increases in density 1 year after the El Niño than medium sites which had lower bleaching levels. This demonstrates a mismatch between bleaching prevalence and mortality levels similar as to what has previously been documented for adult corals [27]. Mechanisms for this have been proposed for adult colonies (e.g., resistance to bleaching but then can only survive temporarily in a bleached state [58,59]; symbiont switching mid heat event [32]), but to our knowledge this pattern has not been documented in juvenile corals. It is possible that juveniles follow some of the same mechanisms as adults but with their different physical structure [20], they may have other mechanisms.

Juvenile corals with a stress-tolerant life history were dominant across the atoll at all time points in this study. While for the entire coral community, Baum et al. [27] also found that stress-tolerant corals dominated Kiritimati’s highly disturbed reefs and all reefs after the 2015-2016 El Niño, prior to the event, competitive corals dominated the lowest disturbance sites. Dominance of stress-tolerant corals was also documented on reefs in the Maldives in the years immediately following both the 1998 [55] and 2016 El Niños [13]. In contrast, after heatwaves other reefs have been dominated by competitive and weedy type corals (e.g., *Acropora* and *Pocillopora*) that colonize open space on reefs and grow rapidly [10,16,57,60]; however, the timescale varies globally, likely due to differences in community dynamics and severity of disturbances. Our study only extended one year past the El Niño and we detected no significant increase in weedy and competitive corals compared to stress-tolerant corals, similar to what was recorded on nearby Jarvis Island [61]. This might indicate that recovery will be suppressed until these corals can contribute significantly to recruitment, as documented on other reefs severely impacted by heat stress [7,10,12,13,62] compared to reefs less impacted by extreme heatwaves [57,63]. Thus, with more studies, it may be possible to roughly calculate recovery times using the severity of the disturbance and composition and density of surviving juvenile corals.

Overall, our study demonstrates the negative impacts that a prolonged heatwave and localized chronic anthropogenic stress had on juvenile corals. This study also highlights differences in impacts amongst juvenile corals with different life history strategies. Given the importance of juvenile corals for reef recovery, continued studies of the impacts of these stressors, the mechanisms by which they affect juvenile corals, and ongoing monitoring of their contributions to reef recovery trajectories is needed.

## Supporting information

S1 Fig Map of reef study sites on Kiritimati (Christmas Island) sampled at each heat stress time point (Before =  2 years before – start (summer 2013, summer 2014, April/May 2015), Early =  2 months (July 2015), Late =  10 months (March 2016), and After =  ~ 1 year after (summer 2017). The sites are divided into five levels of local human disturbance. Village population (red circles) is represented by bubble size.
(TIF)

S2 Fig Representative photos of quadrats surveyed on Kiritimati (a, d) before, (b, e) at the end, and (c, f) after the heatwave on reefs exposed to (a-c) very high levels of local human disturbance and (d-f) very low levels. Photos: Baum Lab, University of Victoria.
(TIF)

S3 Fig Widest width of juvenile corals (n = 7732) on Kiritimati throughout the survey. Mean width was 2.28 cm ( ± 0.01 SE). Bins are 0.5 cm.
(TIF)

S4 Fig Density of juvenile corals with different life history strategies across the human disturbance gradient prior to the 2015-2016 marine heatwave modelled with a two-way interaction. (TIF)

S5 Fig Prevalence of bleaching in juvenile corals across the human disturbance gradient before and during the 2015-2016 marine heatwave modelled with a two-way interaction. (TIF)

S6 Fig Density of juvenile corals across the human disturbance gradient throughout the 2015-2016 marine heatwave modelled with a two-way interaction. (TIF)

S7 Fig Generalized linear mixed model predictor coefficient effect size estimates and 95% confidence intervals for each tested life history strategy: (a) stress-tolerant, (b) competitive, and (c) weedy. In models a and c, human disturbance was modelled as a quadratic (l =  linear, q =  quadratic). Heat stress colors correspond with figure 4. X-axis scale varies among panels.
(TIF)

S8 Fig Density of (a) stress-tolerant, (b) competitive, and (c) weedy juvenile corals across the human disturbance gradient throughout the 2015-2016 marine heatwave modelled with a two-way interaction. (TIF)

S1 Table Number of juvenile coral video quadrats conducted at each of 18 sites around Kiritimati, by expedition before (July 2013, August 2014, May 2015), during (July 2015, March 2016), and after (July 2017) the 2015-2016 El Niño. Sites are ordered first by decreasing levels of local chronic human disturbance then by exposure (Fig 1).
(DOCX)

S2 Table Life history table of juvenile coral taxa identified from video assays processed using Tracker. Coral life history strategy retrieved from the Coral Traits Database (https://coraltraits.org/), unless otherwise noted. Current taxonomy (and name synonymy) retrieved from WoRMS (http://www.marinespecies.org/).
(DOCX)

S3 Table Results for the sensitivity analysis on the heatwave effect by controlling for sites sampled. The first model **(a)** was run using only the 9 sites that were sampled in all four heat stress periods (S1 Fig). The second model **(b)** is run with the 10 sites that were sampled in both the before and late heat stress periods. Bolded values are significantly different from baseline levels (i.e., before, stress-tolerant, leeward) at α =  0.05, asterisks indicate levels of significance ( ⋅ *p* <  0.1, *p <  0.05, **p <  0.01, ***p <  0.001). Red shaded boxes correspond to variables with a negative parameter estimate.
(DOCX)

S4 Table Percent bleaching in individual juvenile (JV) coral colonies (out of a total of n surveyed) at each of three time points relative to the 2015-2016 El Niño (before, early, late) for the top 16 identified coral taxa and two additional species for which there is comparator adult data to compare to. Rank column denotes level of common-ness before the heatwave; the 7^th^ most common was unidentified and thus not included on this table. Adult data was only available for seven species from Baum and colleagues [27]. Species column is colored by life history strategy corresponding to colors used in Fig 3 (red =  stress-tolerant, blue =  competitive, purple =  weedy, tan =  soft coral).
(DOCX)

S5 Table Results for the Wilcoxon matched-pairs signed-rank tests to compare densities of juvenile coral taxa before and after heatwave. Bolded values are significantly different at α =  0.05.
(DOCX)
