# Supplementary figures and images for: Impacts of a prolonged marine heatwave and chronic local human disturbance on juvenile coral assemblages
Source: PLoS One. 2025 Feb 25;20(2):e0300084. doi: 10.1371/journal.pone.0300084 (PMC11856355; doi:10.1371/journal.pone.0300084)

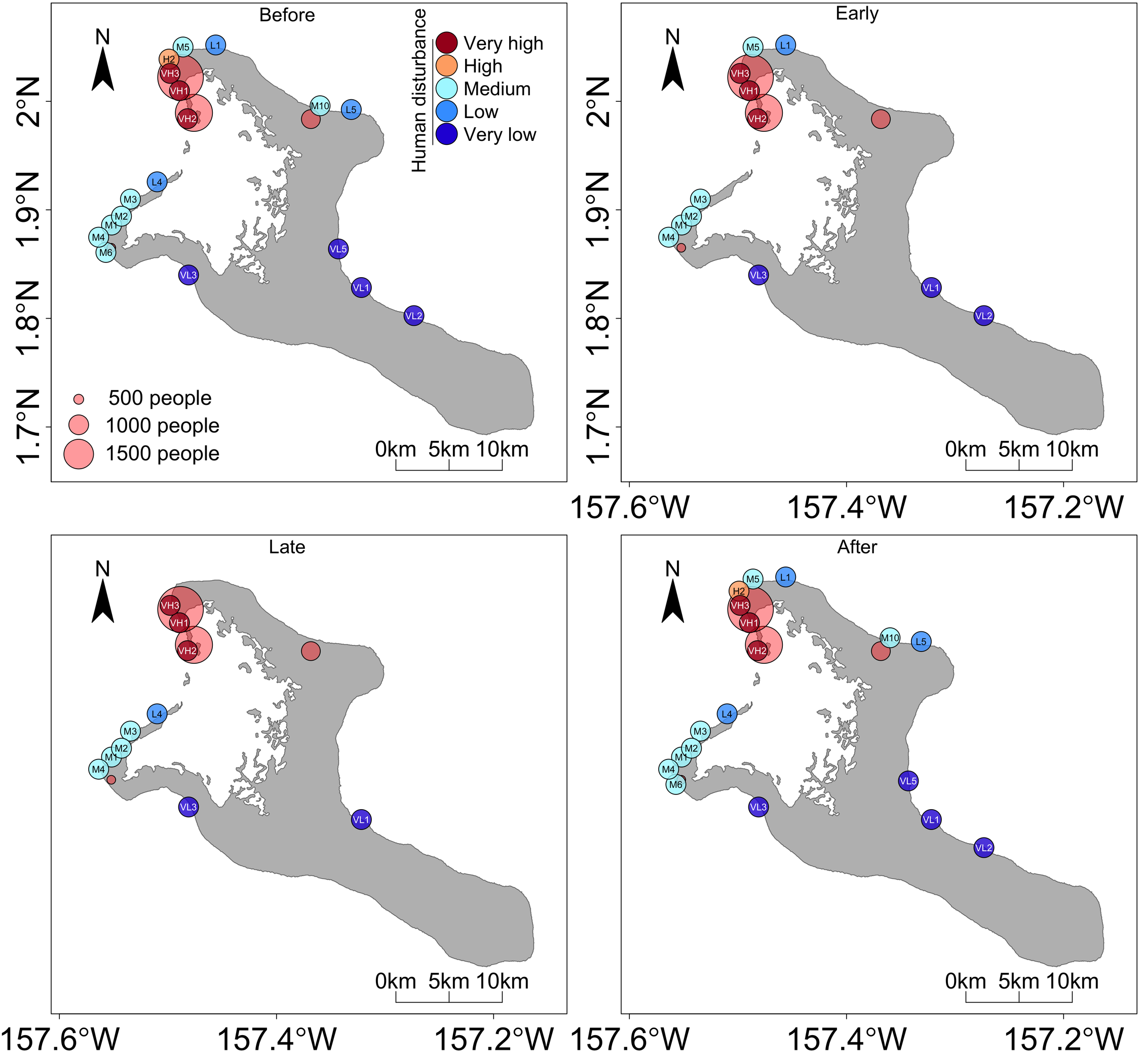

Supplement: S1 Fig — The sites are divided into five levels of local human disturbance. Village population (red circles) is represented by bubble size. (TIF) [file pone.0300084.s001.tif]

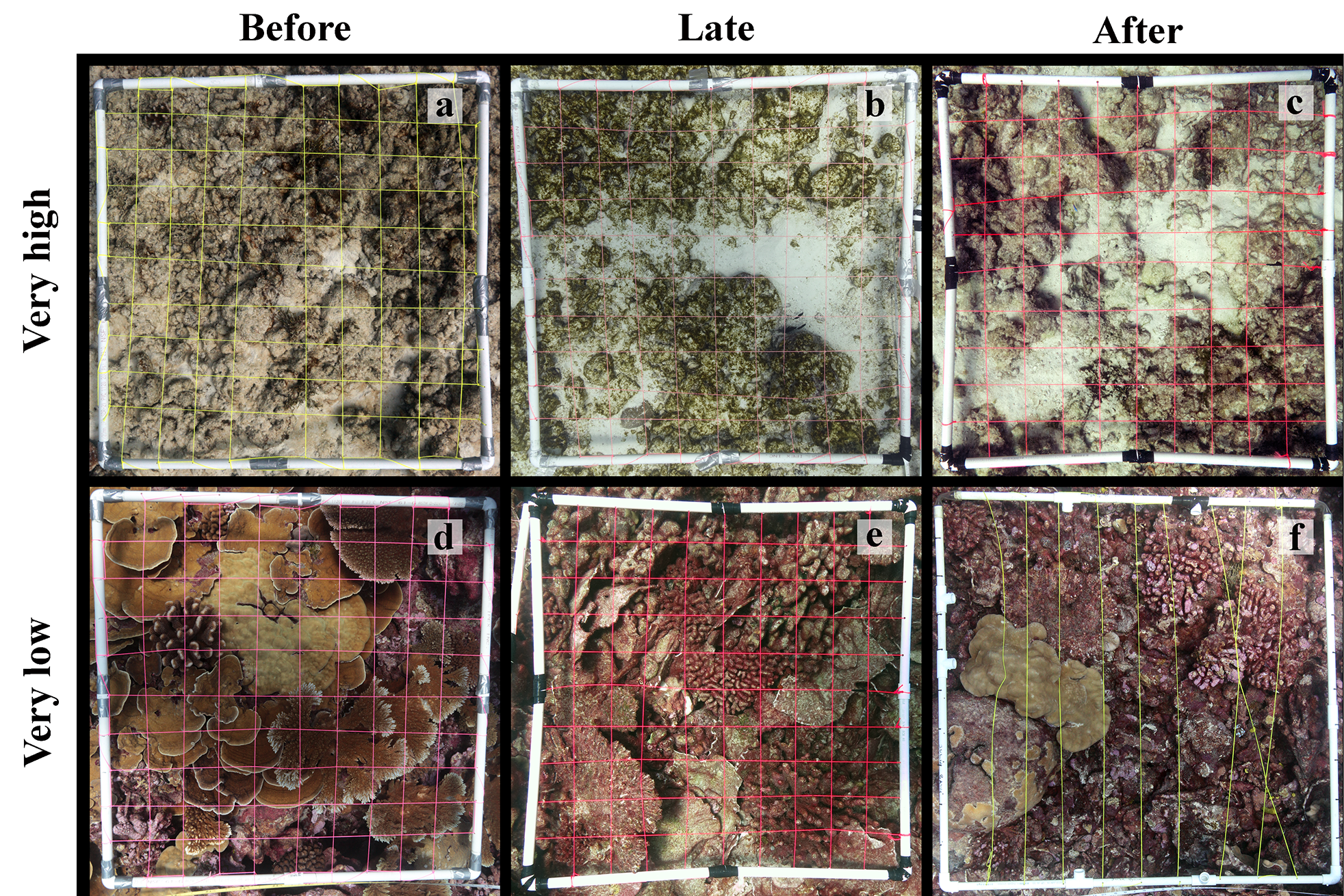

Supplement: S2 Fig — Photos: Baum Lab, University of Victoria. (TIF) [file pone.0300084.s002.tif]

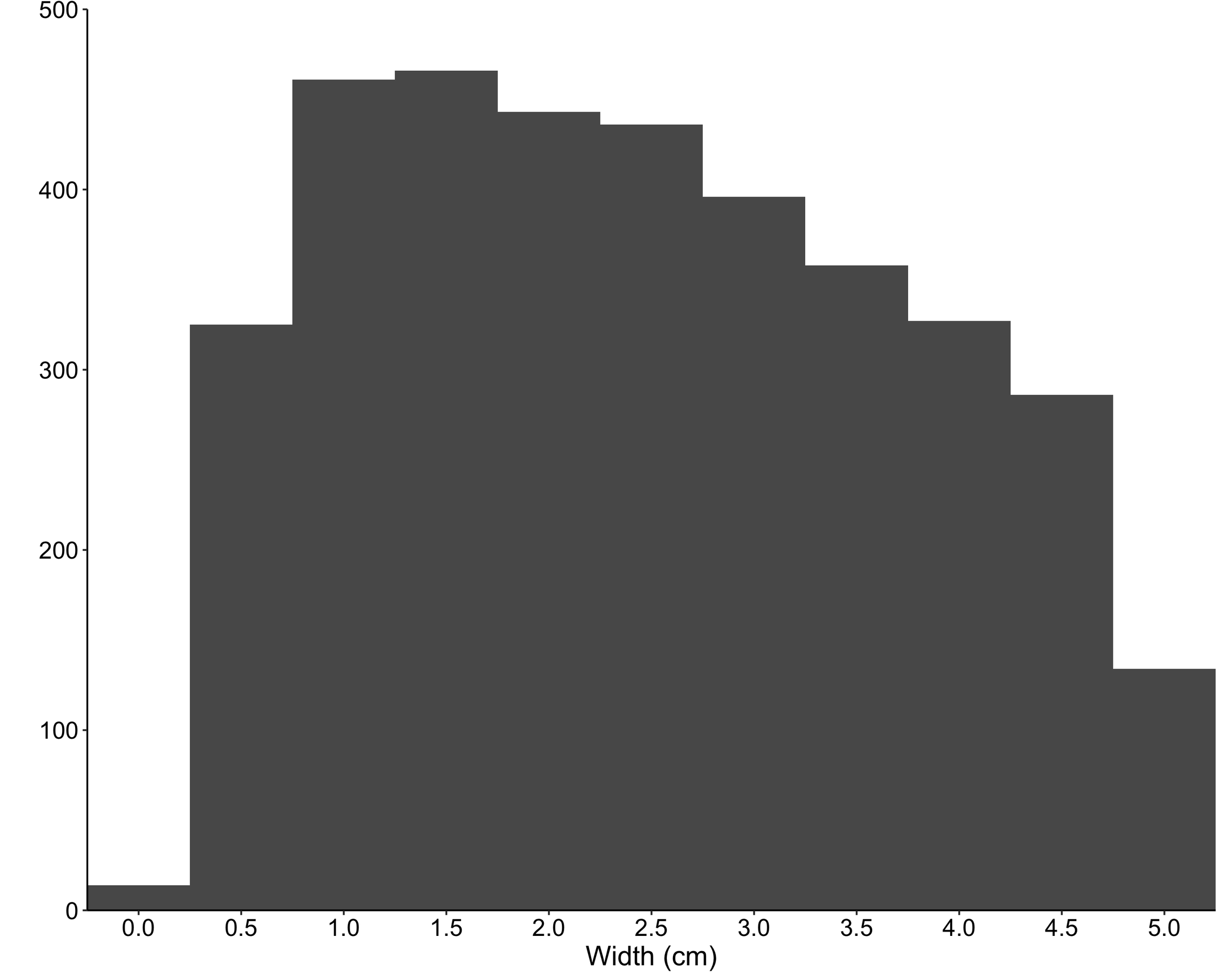

Supplement: S3 Fig — Mean width was 2.28 cm ( ± 0.01 SE). Bins are 0.5 cm. (TIF) [file pone.0300084.s003.tif]

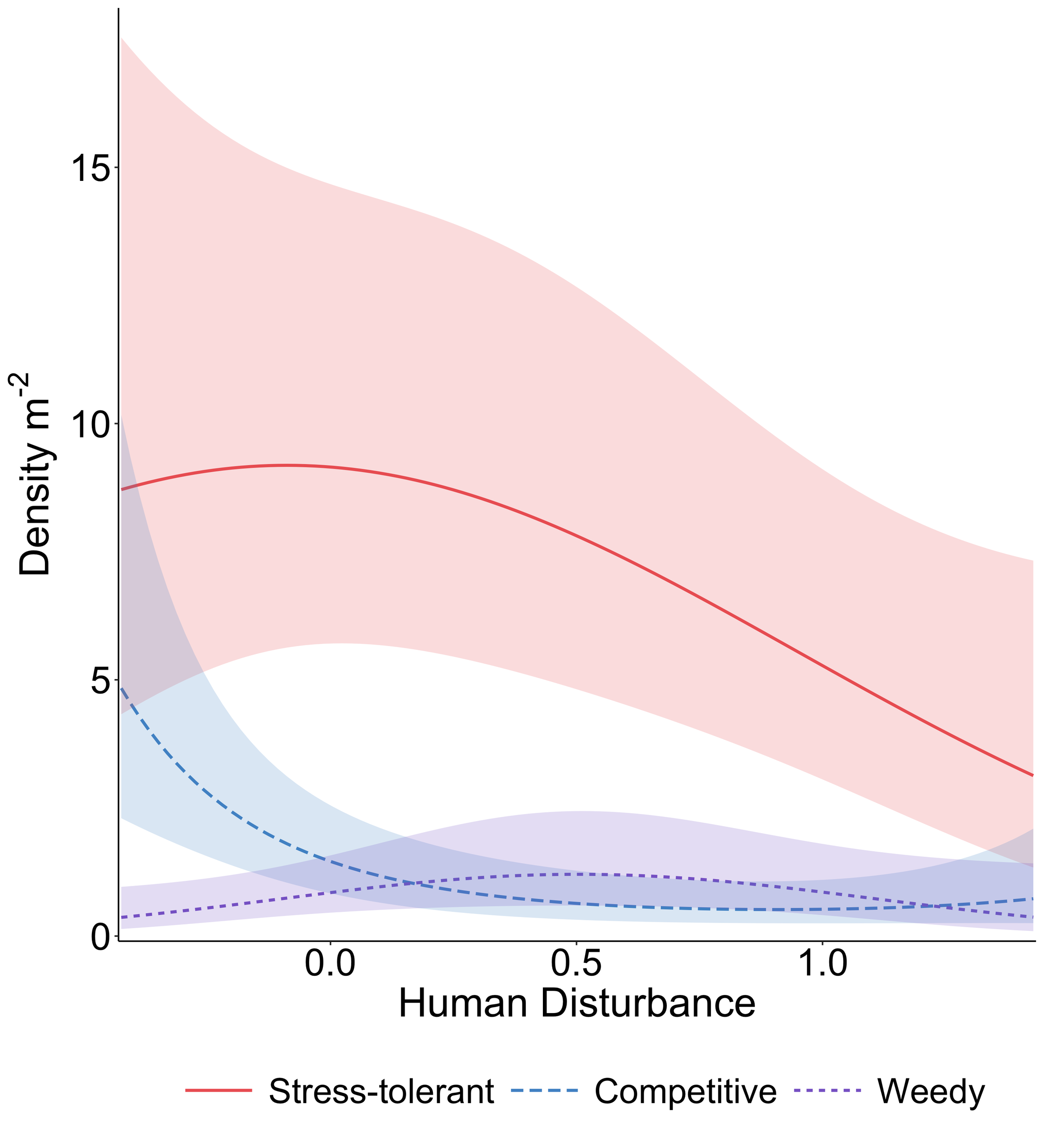

Supplement: S4 Fig — (TIF) [file pone.0300084.s004.tif]

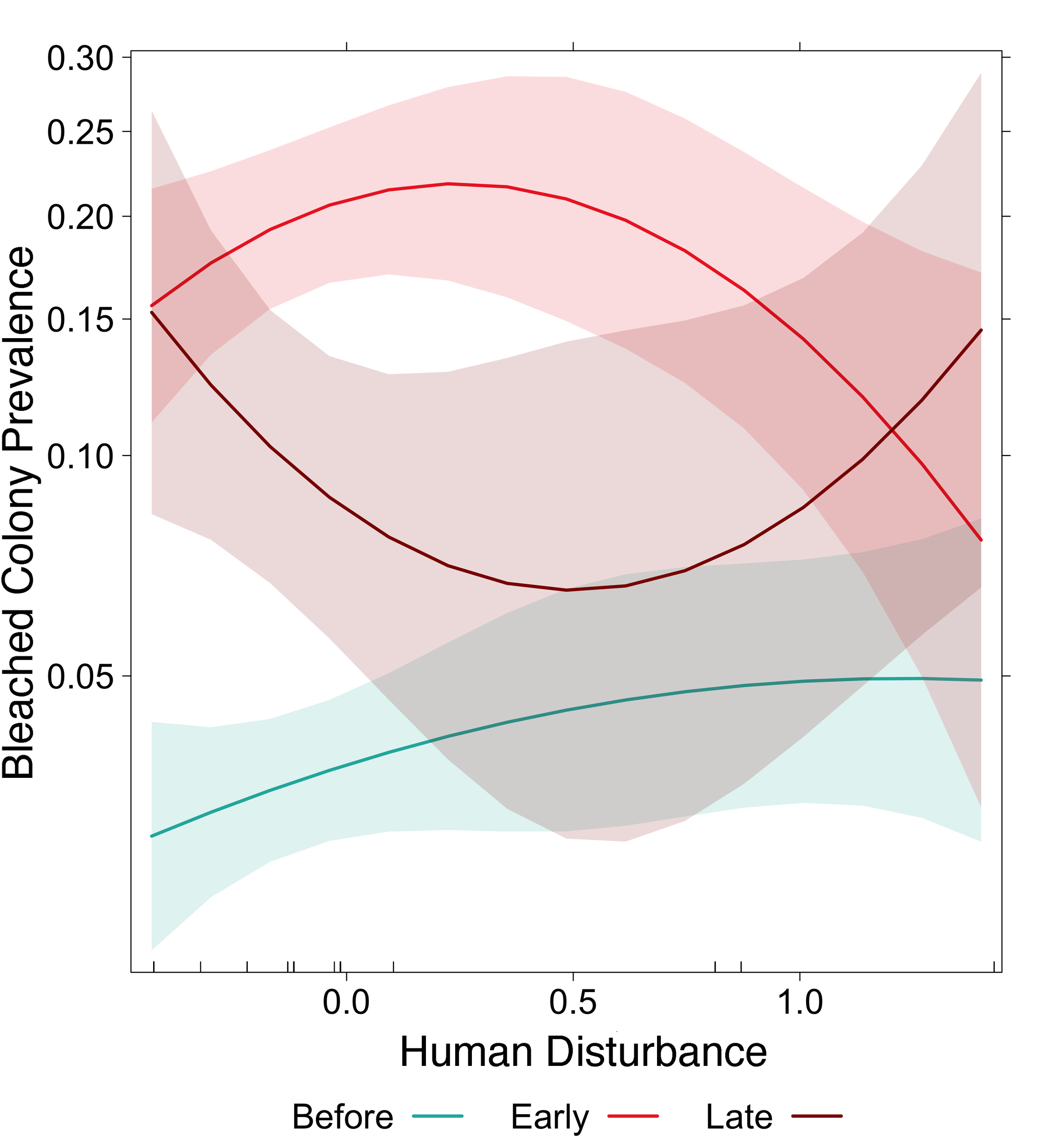

Supplement: S5 Fig — (TIF) [file pone.0300084.s005.tif]

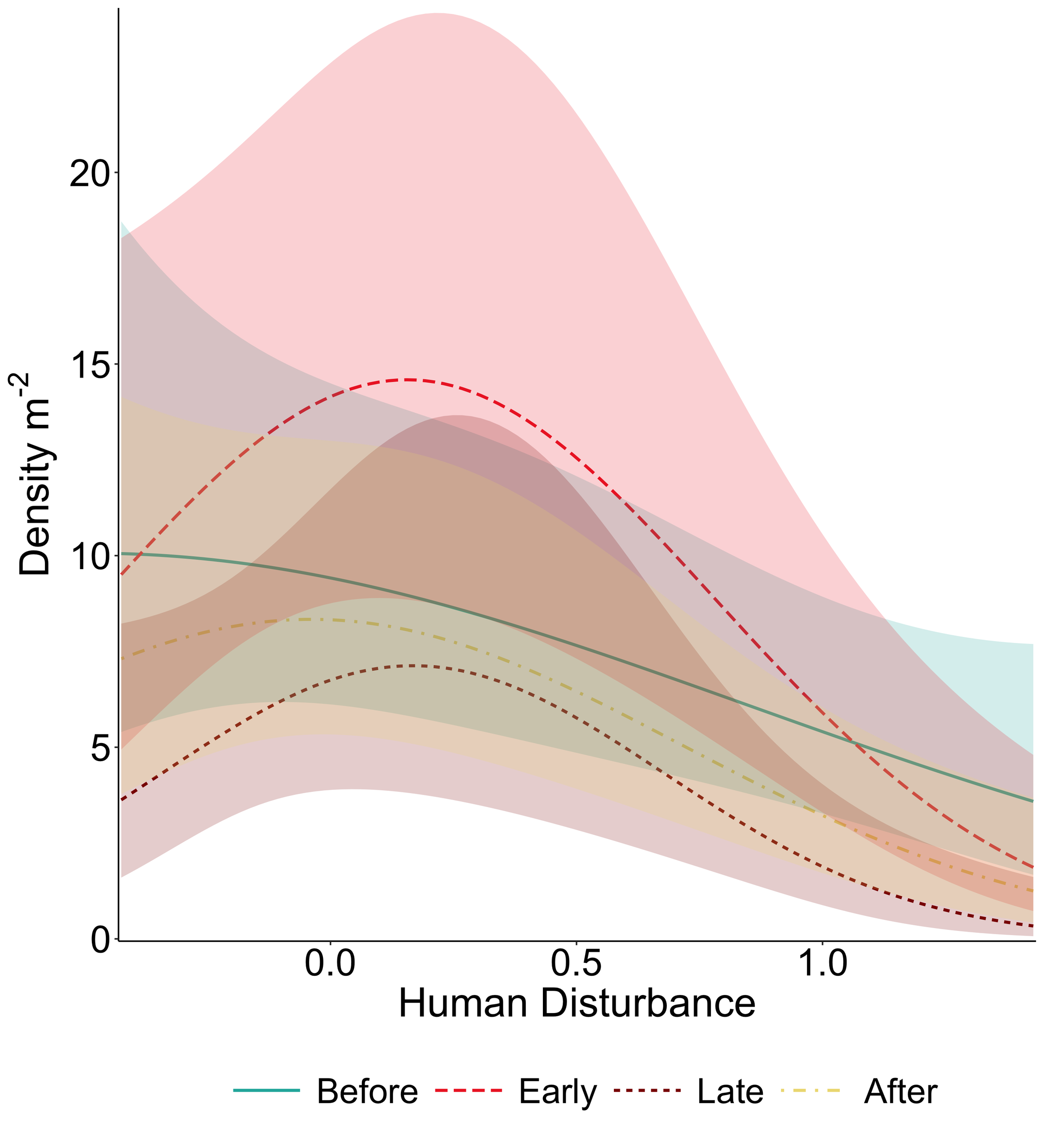

Supplement: S6 Fig — (TIF) [file pone.0300084.s006.tif]

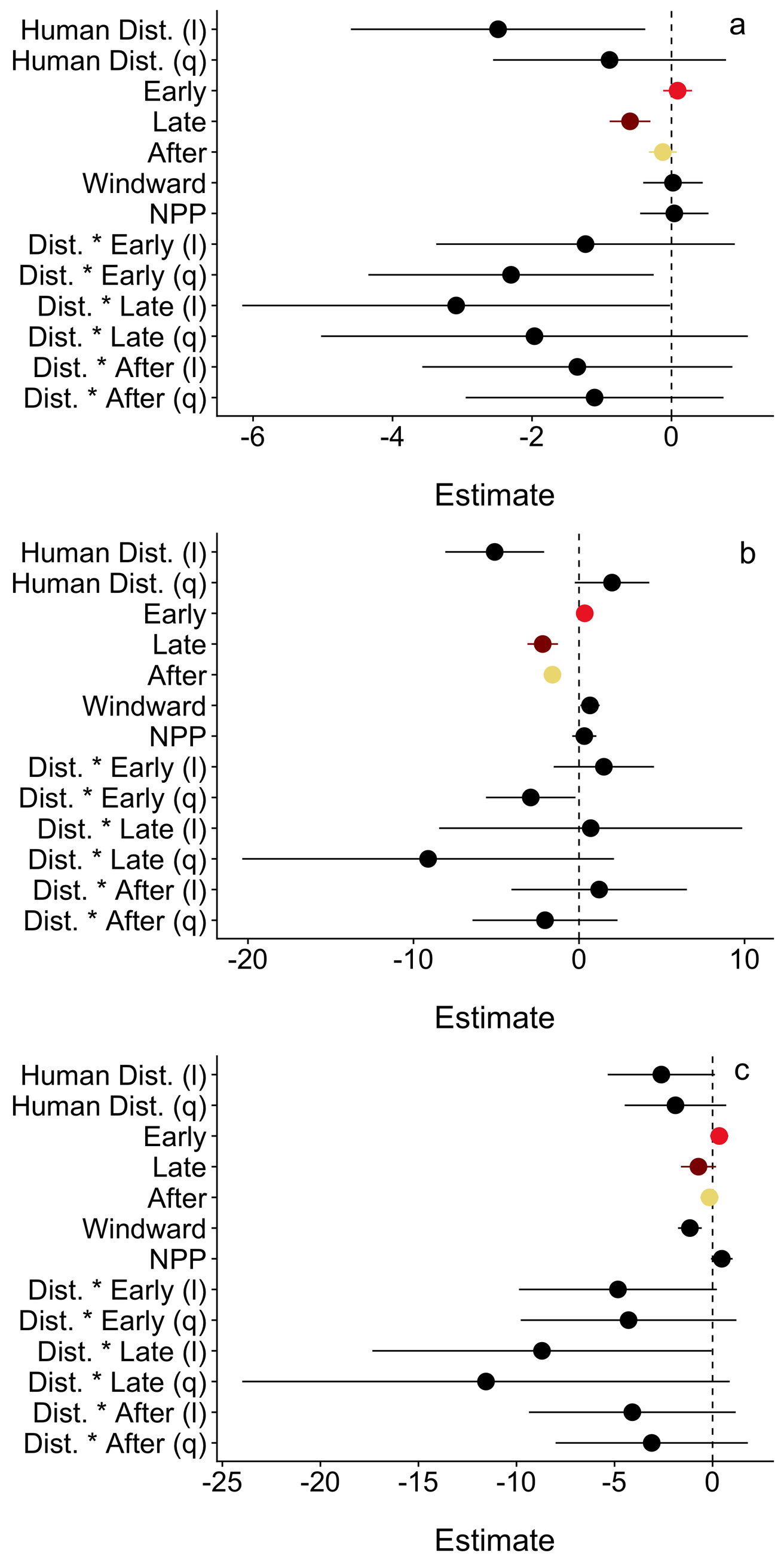

Supplement: S7 Fig — In models a and c, human disturbance was modelled as a quadratic (l = linear, q = quadratic). Heat stress colors correspond with figure 4. X-axis scale varies among panels. (TIF) [file pone.0300084.s007.tif]

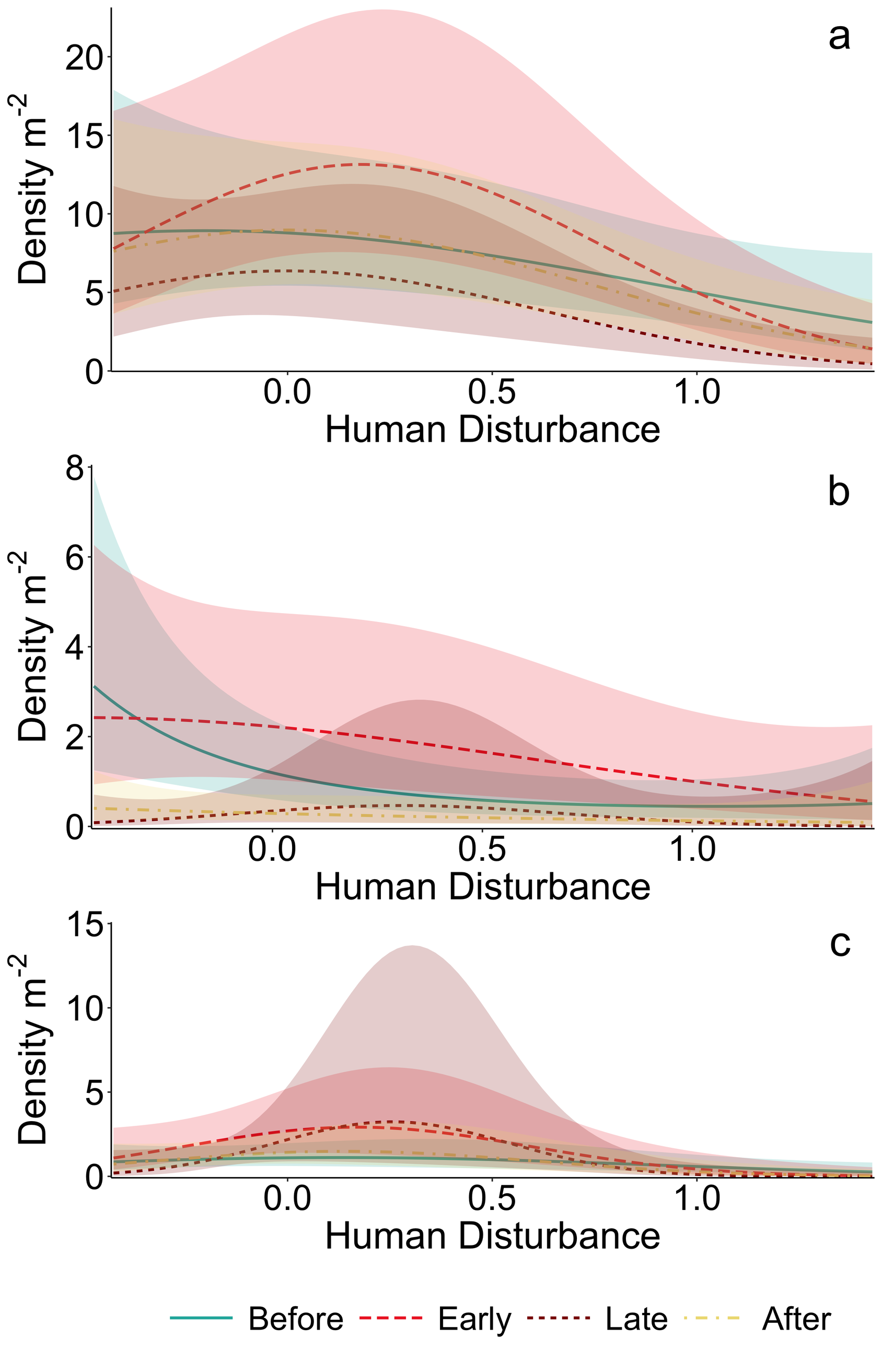

Supplement: S8 Fig — (TIF) [file pone.0300084.s008.tif]
