# Supplementary material for: Impacts of a prolonged marine heatwave and chronic local human disturbance on juvenile coral assemblages
Source: PLoS One. 2025 Feb 25;20(2):e0300084. doi: 10.1371/journal.pone.0300084 (PMC11856355; doi:10.1371/journal.pone.0300084)
Supplement: S1 Table — Sites are ordered first by decreasing levels of local chronic human disturbance then by exposure (Fig 1). (DOCX) [file pone.0300084.s009.docx]

**S1 Table.** **Number of juvenile coral video quadrats conducted at each of 18 sites around Kiritimati, by expedition before (July 2013, August 2014, May 2015), during (July 2015, March 2016), and after (July 2017) the 2015-2016 El Niño.** Sites are ordered first by decreasing levels of local chronic human disturbance then by exposure (Fig 1).

| **Site** | **VH3** | **VH1^a^** | **VH2** | **H2** | **M1** | **M2** | **M3** | **M4** | **M6** | **M5** | **M10** | **L1** | **L5** | **L4** | **VL1** | **VL2** | **VL5** | **VL3** | **Total per time point** |
| --- | --- | --- | --- | --- | --- | --- | --- | --- | --- | --- | --- | --- | --- | --- | --- | --- | --- | --- | --- |
| **Human**  **Disturbance**  **Level** | **VH** | **VH** | **VH** | **H** | **M** | **M** | **M** | **M** | **M** | **M** | **M** | **L** | **L** | **L** | **VL** | **VL** | **VL** | **VL** |  |
| **Exposure** | **LW** | **LW** | **LW** | **LW** | **LW** | **LW** | **LW** | **LW** | **LW** | **WW** | **WW** | **WW** | **WW** | **LW** | **WW** | **WW** | **WW** | **LW** |  |
| **Expedition Date** |  |  |  |  |  |  |  |  |  |  |  |  |  |  |  |  |  |  |  |
| July 2013 | 10 | 9 | 10 | 10 | 10 | 10 | 9 | 1 | 10 | 10 | 10 | N | 10 | 10 | 10 | 4 | 10 | N | 143 |
| August 2014 | 10 | 18 | N | N | 10 | 10 | 10 | 10 | N | 10 | N | 10 | N | N | N | N | N | N | 88 |
| May 2015 | 10 | 10 | 9 | N | 10 | 10 | 10 | N | N | N | N | N | N | N | 10 | N | N | 10 | 79 |
| July 2015 | 10 | 18 | 10 | N | 10 | 10 | 10 | 10 | N | 10 | N | 10 | N | N | 10 | 10 | N | 10 | 128 |
| March 2016 | 9 | 25 | 10 | N | 10 | 10 | 10 | 10 | N | N | N | N | N | 10 | 4 | N | N | 10 | 108 |
| July 2017 | 10 | 17 | 10 | 10 | 10 | 10 | 10 | 10 | 10 | 10 | 10 | 10 | 10 | 9 | 10 | 10 | 10 | 10 | 186 |

N = Not Sampled due to inclement weather; Human disturbance: VH = Very High, H = High, M = Medium, L = Low, VL = Very Low; Exposure: LW = Leeward, WW = Windward

^a^More than 10 videos are done for site VH1 since it is not uncommon for there to be videos of all sand from that highly degraded site.
