# Supplementary material for: Impacts of a prolonged marine heatwave and chronic local human disturbance on juvenile coral assemblages
Source: PLoS One. 2025 Feb 25;20(2):e0300084. doi: 10.1371/journal.pone.0300084 (PMC11856355; doi:10.1371/journal.pone.0300084)
Supplement: S2 Table — Coral life history strategy retrieved from the Coral Traits Database (https://coraltraits.org/), unless otherwise noted. Current taxonomy (and name synonymy) retrieved from WoRMS (http://www.marinespecies.org/). (DOCX) [file pone.0300084.s010.docx]

**S2 Table.** **Life history table of juvenile coral taxa identified from video assays processed using Tracker.** Coral life history strategy retrieved from the Coral Traits Database (<https://coraltraits.org/>), unless otherwise noted. Current taxonomy (and name synonymy) retrieved from WoRMS (<http://www.marinespecies.org/>).

| **Life History** | **Family** | **Species** | **Notes** |
| --- | --- | --- | --- |
| Competitive | Acroporidae | *Acropora* spp. | Includes: corymbose morphology (*A. loripes*  (synonym: *A. rosaria, Madrepora loripes*), *A. subulata*, and hybrids of these species); tabulate morphology (*Acropora hyacinthus* synonym: *M. hyacinthus);* digitate morphology (*A. globiceps* synonym: *M. globiceps*) also includes any corals that could only be identified to genus |
| Competitive | Acroporidae | *Montipora aequituberculata* | *M. aequituberculata* with foliose morphology |
| Competitive | Acroporidae | *Montipora spp.* | *Montipora* spp. with encrusting morphology, includes *M. aequituberculata*, and a few potentially unnamed species |
| Competitive | Pocilloporidae | *Pocillopora* spp. | Includes: *Pocillopora grandis* (synonym: *Pocillopora eydouxi*) and *Pocillopora meandrina*; also includes any corals that could only be identified to genus |
| Competitive^a^ | Pocilloporidae | *Pocillopora* *zelli* |  |
| Competitive^b^ | Dendrophylliidae | *Turbinaria reniformis* |  |
| Stress-tolerant | Acroporidae | *Astreopora* spp. | Includes *A. cucullata, A. myriophthalma*, and *A. suggesta* |
| Stress-tolerant | Agariciidae | *Pavona duerdeni* |  |
| Stress-tolerant | Agariciidae | *Pavona varians* |  |
| Stress-tolerant | Agariciidae | *Gardineroseris planulata* | Synonym: *Agaricia planulata*, *Pavona planulata* |
| Stress-tolerant^c^ | Agariciidae | *Leptoseris mycetoseroides* |  |
| Stress-tolerant | Fungiidae | *Lithophyllon* sp., *Danafungia* spp., *Pleuractis* sp., and *Lobactis* sp., *Cycloseris* sp. (synonym: *Fungia* spp.) | Includes *Lithophyllon concinna* (synonym: *Fungia concinna*), *Danafungia scruposa* (synonym: *F. corona*), *D. horrida* (synonym: *F. danai*), *Pleuractis granulosa* (synonym: *F. granulosa), Lobactis scutaria* (synonym: *F. scutaria*), *C. fragilis* (synonym: *Diaseris fragilis*), and *C. tenuis* (synonym: *F. tenuis*) |
| Stress-tolerant^d^ | Fungiidae | *Herpolitha limax* | Synonym: *Madrepora limax* |
| Stress-tolerant^e^ | Fungiidae | *Sandalolitha robusta* | Synonym: *Podabacia robusta* |
| Stress-tolerant^f^ | Fungiidae | NA | Corals only identifiable to family |
| Stress-tolerant^g^ | Lobophylliidae | *Echinophyllia aspera* | Synonym: *Madrepora aspera* |
| Stress-tolerant^h^ | Lobophylliidae | *Lobophyllia hemprichii* | Synonym: *Manicina hemprichii* |
| Stress-tolerant | Merulinidae | *Favites* spp. | Includes: *Favites pentagona* (synonym: *Madrepora pentagona*) and *Favites halicora* (synonym: *Astraea halicora*); also includes any corals that could only be identified to genus |
| Stress-tolerant | Merulinidae | *Hydnophora microconos* | Synonym: *Monticularia microconos* |
| Stress-tolerant | Merulinidae | *Platygyra* spp. | Includes: *P. daedalea* (synonym: *Madrepora daedalea*), *P. contorta*, *P. ryukyuensis*, and *P. sinensis* (synonym: *Astroria sinensis*); also includes any corals that could only be identified to genus |
| Stress-tolerant | Merulinidae | *Dipsastraea* spp. | Includes: *Dipsastraea matthaii* (synonym: *Favia matthaii)* and *Dipsastraea speciose* (synonym: *Favia speciose*); also includes any corals that could only be identified to genus |
| Stress-tolerant | Merulinidae | *Goniastrea stelligera* | Synonym: *Favia stelligera* |
| Stress-tolerant | Merulinidae | *Astrea* spp. (synonym: *Montastraea* spp.) | May include *A. annuligera* (synonym: *Montastraea* *annuligera*), *A. curta* (synonym: *M.* *curta*); also includes any corals that could only be identified to genus |
| Stress-tolerant^i^ | Merulinidae | NA | Corals only identifiable to family |
| Stress-tolerant | Poritidae | *Porites* spp. | Primarily: *Porites lobata* and may include *P. evermanni* and *P. lutea*; also includes any corals that could only be identified to genus |
| Weedy | Leptastreidae  (synonym: Faviidae) | *Leptastrea* spp. | Includes *L. pruinose, L. bewickensis* and *L. purpurea* (synonym: *Astaea purpurea*); also includes any corals that could only be identified to genus |
| Generalist | Merulinidae | *Hydnophora exesa* | Synonym: *Madrepora exesa* |
| Unknown | Coscinaraeidae | *Coscinaraea* spp. | No recorded life history strategy for any species in the family |
| Unknown | Dendrophylliidae | *Turbinaria stellulata* | Could not be extracted from congenerics; Synonym: *Astrea stellulata* |
| Unknown | Psammocoridae | *Psammocora profundacella* | No recorded life history strategy for any accepted species in the family |

^a^Life history strategy extracted from congeneric *Pocillopora eydouxi*

^b^Life history strategy extracted from congeneric *Turbinaria mesenterina*

^c^Life history strategy extracted from family Agariciidae (i.e., *Gardineroseris* and *Pavona*)

^d^Life history strategy extracted from family Fungiidae (i.e., *Fungia*)

^e^Life history strategy extracted from family Fungiidae (i.e., *Fungia*)

^f^All corals with a known life history in family Fungiidae on Kiritimati have the same life history strategy so extracted to the family level identifications

^g^Life history strategy extracted from congeneric *Echinophyllia orpheensis*

^h^Life history strategy extracted from congenerics *Lobophyllia corymbosa* and *Lobophyllia pachysepta*

^i^As *Hydnophora exesa*, the only non-stress-tolerant species in the family Merulinidae on Kiritimati, is morphometrically distinct, corals that could only be identified to family were assigned stress-tolerant due to the probably that they were not *H. exesa*
