# Supplementary material for: Impacts of a prolonged marine heatwave and chronic local human disturbance on juvenile coral assemblages
Source: PLoS One. 2025 Feb 25;20(2):e0300084. doi: 10.1371/journal.pone.0300084 (PMC11856355; doi:10.1371/journal.pone.0300084)
Supplement: S3 Table — The first model (a) was run using only the 9 sites that were sampled in all four heat stress periods (S1 Fig). The second model (b) is run with the 10 sites that were sampled in both the before and late heat stress periods. Bolded values are significantly different from baseline levels (i.e., before, stress-tolerant, leeward) at α = 0.05, asterisks indicate levels of significance ( ⋅ p < 0.1, *p < 0.05, **p < 0.01, ***p < 0.001). Red shaded boxes correspond to variables with a negative parameter estimate. (DOCX) [file pone.0300084.s011.docx]

**S3 Table. Results for the sensitivity analysis on the heatwave effect by controlling for sites sampled.** The first model **(a)** was run using only the 9 sites that were sampled in all four heat stress periods (S1 Fig). The second model **(b)** is run with the 10 sites that were sampled in both the before and late heat stress periods. Bolded values are significantly different from baseline levels (i.e., before, stress-tolerant, leeward) at α = 0.05, asterisks indicate levels of significance (⋅ *p* < 0.1*,* * *p* < 0.05, ** *p* < 0.01, *** *p* < 0.001). Red shaded boxes correspond to variables with a negative parameter estimate.

|  | | **(a) Only sites sampled in all four heat stress periods** | **(b) Only sites sampled in both before & late periods** |
| --- | --- | --- | --- |
| Human disturbance | Linear | **-5.499 ± 1.97**** | -3.572 ± 1.858 ⋅ |
|  | Quad. | -0.943 ±1.579 | -1.730 ± 1.665 |
| Heat stress | Early | 0.148 ± 0.108 | 0.161 ± 0.106 |
|  | Late | **-0.839 ± 0.165***** | **-0.825 ± 0.159***** |
|  | After | **-0.360 ± 0.129**** | **-0.356 ± 0.125**** |
| HD * HS (Early) | Linear | -2.624 ± 0.582 ⋅ | -2.660 ± 1.601 ⋅ |
|  | Quad. | **-4.387 ± 1.532**** | **-4.372 ± 1.541**** |
| HD * HS (Late) | Linear | **-5.304 ± 2.488*** | **-5.262 ± 2.496*** |
|  | Quad. | **-5.101 ± 2.450*** | **-5.141 ± 2.477*** |
| HD * HS (After) | Linear | -3.275 ± 1.769 | -3.231 ± 1.786 ⋅ |
|  | Quad. | 0.537 ± 1.633 ⋅ | 0.584 ± 1.656 |
| Life history | Competitive | **-1.878 ± 0.122***** | **-1.897 ± 0.121***** |
|  | Weedy | **-2.058 ± 0.129***** | **-2.002 ± 0.125***** |
| Exposure | Windward | -0.039 ± 0.379 | 0.004 ± 0.422 |
| Net Primary Productivity | | 0.407 ± 0.371 | -0.006 ± 0.331 |

*Quad.* = Quadratic relationship; *HD* = Human disturbance; *HS* = Heat stress
