# Supplementary material for: Impacts of a prolonged marine heatwave and chronic local human disturbance on juvenile coral assemblages
Source: PLoS One. 2025 Feb 25;20(2):e0300084. doi: 10.1371/journal.pone.0300084 (PMC11856355; doi:10.1371/journal.pone.0300084)
Supplement: S4 Table — Rank column denotes level of common-ness before the heatwave; the 7th most common was unidentified and thus not included on this table. Adult data was only available for seven species from Baum and colleagues [27]. Species column is colored by life history strategy corresponding to colors used in Fig 3 (red = stress-tolerant, blue = competitive, purple = weedy, tan = soft coral). (DOCX) [file pone.0300084.s012.docx]

**S4 Table. Results for the Wilcoxon matched-pairs signed-rank tests to compare densities of juvenile coral taxa before and after heatwave.** Bolded values are significantly different at α = 0.05.

| **Taxa** | ***T* statistic** | **P-value** |
| --- | --- | --- |
| *L. mycetoseroides* | 613.5 | 0.281 |
| *P. varians* | 725 | 0.089 |
| ***M. aequituberculata*** | **514** | **<0.0001** |
| ***G. planulata*** | **712** | **0.029** |
| *P. lobata* | 729 | 0.149 |
| *H. microconos* | 688 | 0.055 |
| **Unidentifiable** | **727** | **0.042** |
| **Fungiidae family spp.** | **542** | **0.034** |
| *Leptastrea* spp. | 560 | 0.096 |
| *D. matthaii* | 626 | 0.066 |
| *Acropora* spp. | 198 | 0.348 |
| *Lobophytum* spp. | 26 | 0.050 |
| *G. stelligera* | 475 | 0.385 |
| ***Montipora* (encrusting)** | **407** | **<0.0001** |
| ***Pocillopora* spp.** | **437** | **0.005** |
| *A. subulate* | 6 | 0.181 |
| *Platygyra* spp. | 337 | 0.956 |
| ***P. meandrina*** | **239** | **0.002** |
| *Astrea* spp. | 106 | 0.379 |
| *D. speciosa* | 200 | 0.155 |
| *P. zelli* | 1 | 1 |
| ***F. halicora*** | **234** | **0.015** |
| *E. aspera* | 5 | 0.423 |
| *A. curta* | 92 | 0.919 |
| *L. pruinose* | 104 | 0.428 |
| *F. pentagona* | 274 | 0.100 |
| ***P. grandis*** | **36** | **0.013** |
| *Dipsastraea* spp. | 104 | 0.057 |
| *A. cucullata* | 1 | 1 |
| *Cladiella* spp. | 4 | 0.773 |
| *T. stellulata* | 1.5 | 1 |
| *A. loripes* | 7.5 | 1 |
| *L. purpurea* | 37.5 | 0.936 |
| *M. platyphylla* | 3 | 0.371 |
| *P. duerdeni* | 32 | 0.262 |
| *P. profundacella* | 17.5 | 1 |
| *Coscinaraea* spp. | 10 | 0.572 |
| *H. exesa* | 4 | 0.773 |
| *Sarcophyton* spp. | 12 | 0.233 |
| ***Astreopora* spp.** | **32.5** | **0.040** |
| *H. limax* | 3 | 0.371 |
| *L. bewickensis* | 2 | 0.773 |
| *A. globiceps* | 1 | 1 |
| *Montipora* spp. | 1.5 | 1 |
| *A. rosaria-subulata* | 0 | 1 |
| *L. hemprichii* | 0 | 0.174 |
