# Supplementary material for: Impacts of a prolonged marine heatwave and chronic local human disturbance on juvenile coral assemblages
Source: PLoS One. 2025 Feb 25;20(2):e0300084. doi: 10.1371/journal.pone.0300084 (PMC11856355; doi:10.1371/journal.pone.0300084)
Supplement: S5 Table — Bolded values are significantly different at α = 0.05. (DOCX) [file pone.0300084.s013.docx]

**S5 Table. Percent bleaching in individual juvenile (JV) coral colonies (out of a total of n surveyed) at each of three time points relative to the 2015-2016 El Niño (before, early, late) for the top 16 identified coral taxa and two additional species** for which there is comparator adult data**.** Rank column denotes level of juvenile density common-ness before the heatwave; the 7^th^ most common was unidentified and thus not included on this table. Adult data was only available for seven species from Baum and colleagues [27]. Species column is colored by life history strategy corresponding to colors used in figure 3 (red = stress-tolerant, blue = competitive, purple = weedy, tan = soft coral).

| **Species** | **Rank** | **Before** | | **Early** | | **Late** | |
| --- | --- | --- | --- | --- | --- | --- | --- |
|  |  | **JV Corals** | **Adults** | **JV Corals** | **Adults** | **JV Corals** | **Adults** |
| *L. mycetoseroides* | 1 | 3.4% (n=531) |  | 25.3% (n=186) |  | 9.8% (n=51) |  |
| *P. varians* | 2 | 0.5% (n=421) |  | 7.5% (n=199) |  | 0% (n=55) |  |
| *M. aequituberculata* | 3 | 2.1% (n=328) | 1.1% (n=93) | 3.4% (n=145) | 22.2% (n=90) | 0 recorded | 100% (n=3) |
| *G. planulata* | 4 | 4.7% (n=363) |  | 8.8% (n=125) |  | 1.6% (n=63) |  |
| *P. lobata* | 5 | 6% (n=301) | 2.4% (n=85) | 8.4% (n=179) | 8.4% (n=83) | 6.7% (n=104) | 79.4% (n=34) |
| *H. microconos* | 6 | 4.3% (n=257) | 5.9% (n=34) | 11.9% (n=109) | 36.4% (n=33) | 0% (n=22) | 57.1% (n=7) |
| *Fungiidae family* spp. | 8 | 21.9% (n=137) |  | 33.3% (n=57) |  | 66.7% (n=9) |  |
| *Leptastrea* spp. | 9 | 11% (n=181) |  | 6.5% (n=92) |  | 13.1% (n=69) |  |
| *Dipsastraea* spp. | 10 | 10.9% (n=229) | 3.8% (n=53) | 38.4% (n=86) | 59% (n=39) | 17.5% (n=40) | 44.4% (n=9) |
| *Acropora* spp. | 11 | 0% (n=74) |  | 18.8% (n=48) |  | 0% (n=5) |  |
| *Lobophytum* spp*.* | 12 | 0% (n=21) |  | 0% (n=6) |  | 0 recorded |  |
| *G. stelligera* | 13 | 6.7% (n=104) |  | 44.7% (n=38) |  | 7.7% (n=13) |  |
| *Montipora* (encrusting) | 14 | 7.5% (n=93) |  | 12.5% (n=72) |  | 0% (n=3) |  |
| *Pocillopora* spp. | 15 | 13% (n=69) |  | 3.8% (n=26) |  | 20% (n=5) |  |
| *A. subulata* | 16 | 20% (n=10) |  | 11.5% (n=26) |  | 0 recorded |  |
| *Platygyra* spp. | 17 | 18.1% (n=72) | 15.4% (n=39) | 20% (n=45) | 63.6% (n=33) | 27.3% (n=22) | 33.3% (n=18) |
| *F. pentagona* | 26 | 6.3% (n=32) | 20.5% (n=39) | 12.5% (n=8) | 68.6% (n=35) | 0% (n=2) | 40% (n=10) |
| *P. grandis* | 27 | 7.7% (n=13) | 9.7% (n=93) | 0% (n=6) | 9.1% (n=88) | 0 recorded | 100% (n=1) |
